# RSV: perspectives to strengthen the need for protection in all infants

**DOI:** 10.1186/s12982-021-00104-5

**Published:** 2021-10-21

**Authors:** Jose Antonio Navarro Alonso, Louis J. Bont, Elena Bozzola, Egbert Herting, Federico Lega, Silke Mader, Marta C. Nunes, Octavio Ramilo, George Valiotis, Catherine Weil Olivier, Ann Yates, Saul N. Faust

**Affiliations:** 1 Expert of the Spanish and European Medicines Agency, Amsterdam, The Netherlands; 2 Wilhelmina Children’s Hospital and UMC Utrecht, Utrecht, The Netherlands; 3 Pediatric and Infectious Diseases Unit, Bambino Gesù Children Hospital, Rome, Italy; 4 Department of Pediatrics, University of Lübeck, Lübeck, Germany; 5 Centre for Research on Healthcare Management, Bocconi University, Milan, Italy; 6 European Foundation for the Care of Newborn Infants (EFCNI), Munich, Germany; 7 Department of Science and Technology/National Research Foundation: Vaccine Preventable Diseases Unit, Medical Research Council: Respiratory and Meningeal Pathogens Research Unit, School of Pathology, Faculty of Health Sciences, University of the Witwatersrand, Johannesburg, South Africa; 8 Center for Vaccines and Immunity, The Abigail Wexner Research Institute at Nationwide Children’s Hospital, Room WA4021, 700 Children’s Drive, Columbus, OH 43205 USA; 9 European Health Management Association (EHMA), Brussels, Belgium; 10 University of Paris, 7 Denis Diderot, 28 rue Parmentier, Neuilly sur Seine, 92200 Paris, France; 11 International Confederation of Midwives (ICM), The Hague, The Netherlands; 12 Faculty of Medicine and Institute for Life Sciences, University of Southampton, Hampshire, Southampton, UK; 13 NIHR Southampton Clinical Research Facility and NIHR Southampton Biomedical Research Centre, University Hospital Southampton NHS Foundation Trust, Hampshire, Southampton, UK

**Keywords:** Global childhood disease burden, Infant and child health, Infant mortality, RSV, Respiratory syncytial virus, RSV-ALRI, RSV-associated acute lower respiratory illness, Vaccine

## Abstract

Respiratory syncytial virus (RSV)—the most common viral cause of bronchiolitis—is a significant cause of serious illness among young children between the ages of 0–5 years and is especially concerning in the first year of life. Globally, RSV is a common cause of childhood acute lower respiratory illness (ALRI) and a major cause of hospital admissions in young children and infants and represents a substantial burden for health-care systems. This burden is strongly felt as there are currently no effective preventative options that are available for all infants. However, a renaissance in RSV prevention strategies is unfolding, with several new prophylactic options such as monoclonal antibodies and maternal vaccinations that are soon to be available. A key concern is that health decision makers and systems may not be ready to take full advantage of forthcoming technological innovations. A multi-stakeholder approach is necessary to bridge data gaps to fully utilise upcoming options. Knowledge must be made available at multiple levels to ensure that parents and doctors are aware of preventative options, but also to ensure that stakeholders and policymakers are given the necessary information to best advise implementation strategies.

## Background

RSV is one of the most common causes of childhood acute lower respiratory illness, nearly 100% of children are infected within the first two years of life. Global estimates from 2015 reported that there were 33 million episodes of RSV-ALRI that resulted in about three million hospital admissions and 59,600 in-hospital deaths in children under 5 years old [1]. At present, there are no effective preventative options for all infants. Most infants and children infected with acute lower respiratory illness are otherwise healthy, suggesting that preventive measures for all infants, rather than targeting those considered at risk, will be a more effective strategy. Raising the awareness of a multi-stakeholder group including policy makers and parents of preventive approaches to RSV as well as health professionals, service providers and donors is essential if we are to tackle the burden of child and infant RSV-ALRI death and disease. The article is based on the perspectives gathered from the RSV Expert Group Event: RSV Prevention for all Infants, held on Tuesday, September 29, 2020, that brought together thirteen global experts comprising clinical experts working on RSV, midwives’ organisations, parents’ association groups and health management experts to discuss preventive strategies regarding childhood immunisation against RSV.

### Burden of disease

Nearly 100% of children are infected with RSV in the first 2 years of life [2]. Ten percent will go to see a doctor and, of these, one in ten will require hospitalisation [3]. Today, an unacceptably large number of infants suffer from severe respiratory illness due to RSV. Importantly, most hospitalisations for RSV occur in otherwise healthy infants born at term [4]. RSV infections are also responsible for a large outpatient healthcare burden and might lead to long-term complications such as wheeze or asthma. Globally, RSV is the most significant cause of ALRI-related death in the first year of life [5]. Although disease surveillance and data-gathering for RSV infection have often not been prioritised, global estimates from 2015 reported that there were 33 million episodes of RSV-ALRI that resulted in about three million hospital admissions [6] and 59,600 in-hospital deaths in children under 5 years old. In children under 6 months of age, 1.4 million hospital admissions and 27,300 deaths in the hospital were due to RSV-ALRI. There were 1,20,000 deaths overall, at least 50% of which were in infants younger than 5 months [7].

### The economic burden of RSV illness

RSV is a leading cause of hospitalisation in infants and RSV-related morbidity results in a substantial number of emergency department and outpatient visits. This is translated into significant economic costs and burden that could be reduced and even avoided if effective prevention strategies of RSV infection were implemented. A recent publication from Italy, confirms the high hospitalisation costs associated with bronchiolitis [8]. Where the aetiology was linked to RSV, the costs were, on average, around 6% higher compared to bronchiolitis by other aetiologies. This is due to the longer hospitalisation and more frequent admissions to the intensive care unit of RSV patients. Age at admission of less than 3 months was found to be a risk factor for severe bronchiolitis [1].

### The RSV prevention landscape—emerging opportunities

At present, the only approved agent for RSV prophylaxis is the anti-RSV monoclonal antibody (mAbs) palivizumab [Synagis(R), AbbVie], which is utilised for passive immunoprophylaxis of various high-risk infants predisposed to developing severe RSV disease and must be injected monthly throughout the RSV season. It is licensed in numerous countries and available for use in pre-term infants and infants with chronic lung disease or congenital heart disease (CLD/CHD).

Palivizumab has demonstrated efficacy as a prophylactic treatment of RSV [9] and operates by binding to the RSV F protein, which is required for viral and host membrane fusion. Therefore, palivizumab binding to RSV diminishes the capacity of RSV to enter the host cells, conferring a degree of protection from infection.

New long-acting mAbs that have been designed to provide protection with a single injection given early after birth or prior to the start of the season are also in clinical development.

Maternal immunisation (MI) has been proposed as an alternative option, with studies suggesting a vaccine during the last trimester of pregnancy could increase the amount of RSV antibodies passed from the mother to the infant, providing protection against RSV for the initial weeks or months of life [10]. However, while several developments and RSV clinical trial programmes are continuing, RSV maternal immunisation is not currently licensed anywhere.

This commentary is focused on understanding the RSV prevention landscape with a specific focus on options that may soon be available to prevent RSV infection from birth in all infants.

### Key insights to strengthen current RSV response

Technological innovations seem likely to deliver several interventions to prevent RSV morbidity and mortality in the first year of life. The Roundtable concluded that major changes are needed in both societal attitudes and health systems if these new technologies are to achieve their full potential.

There is a need to move to an all-infants prevention strategy: RSV is recognised as a serious respiratory illness that can cause high morbidity and mortality among infants at particularly high risk, but most infants hospitalised with severe RSV infection were born at term and were healthy infants until infected [11–13]. This has weighty implications for any prevention mechanism, and only the protection of all infants may achieve the most significant public health impact.

RSV infection has a well-defined seasonality in temperate countries: the infection and the burden of illness that results are crowded into just a few months. This results in pressure on emergency services, wards, and paediatric hospital capacity. Ideally, a prevention tool would protect those infants born in the season or prior to the season who are vulnerable during that season. It may be that the programmatic challenges of identifying infants falling in any criteria to define those most at risk, together with changing hospital procedures to accommodate such definitions and the as-yet-unknown impact of COVID-19 could result in all infants being offered some form of RSV prevention.

Recent advances in RSV prevention strategies hold great promise but require careful planning: both long-acting/high affinity mAbs and maternal vaccines/maternal immunisation are both likely to be useful instruments for the prevention of RSV infection. Both provide passive immunisation rather than the alternative of using an active RSV vaccine given to infants that would not protect them in the first weeks of life.

The MI approach is useful, and it is familiar to the health system/medical community and the healthcare providers. In several countries, there is a strong body of experience on MI to protect infants in their first weeks of life against other infectious diseases [12]. Delivery platforms already exist to offer protection against tetanus, influenza and more recently against pertussis. But there are also challenges with MI. It is unknown whether MI would offer protection for more than 12–16 weeks. There is a theoretical concern that RSV antibodies might, as is documented in MI against pertussis, interfere with the infant’s response to vaccination during the early stages of life, in a process referred to as blunting, or immune interference [14].

Many mothers may also refuse a maternal vaccine, as many do with influenza vaccines—in France, for example, the experience with MI for influenza has been poor [15]. Information regarding the safety profile of maternal immunisation for breastfeeding mothers may be useful in improving perception of the vaccination, and therefore in increasing uptake. There is also some concern regarding the co-administration of a potential RSV vaccine along with other maternal vaccines so there would need to be strong calendar-based management of vaccines for pregnant women [16]. The last trimester of pregnancy should be the right time to administer an RSV vaccine, but this must be confirmed in clinical trials.

Passive immunisation using mAbs is administered directly to the infant immediately after birth when born during the RSV season or any time before the RSV season for infants born out of the season. As noted above, the antibodies diminish the capacity of RSV to enter cells. Clinical trial data suggest that the new mAbs can offer effective protection. One injection may protect against RSV infection for a typical 5-month RSV season [2]. Because routine passive immunisation for all infants using mAbs is a new approach for protection, there are challenges in how mAbs are regulated and approved for use within health systems: they may be viewed in the same product category as vaccines, but the regulations in place may not permit this. It is possible that COVID-19 response strategies could help increase acceptance because there is research ongoing with multiple COVID-19 mAbs as a preventative approach. A mAb immunisation administered just after birth for infants born during the RSV season would require all health professionals involved in pregnancy and delivery to be involved in education and support of parents. Midwives are likely to be particularly important in this and would require training.

The use of new prevention tools will require education. Because the threat from RSV is insufficiently understood by parents and even underestimated by some health professionals, it may be more difficult to introduce new prevention strategies for all infants. More time and effort must be dedicated to informing and educating clinicians, care givers, communities, and policy makers on the burden of RSV infection and the importance of an effective RSV prevention strategy [8].

### A roadmap for the future

New preventive options are likely to be available soon—particularly mAbs. The group developed a set of actions to be included in a near-term plan of action to prepare an enabling environment for future passive immunisation product launches.

Data gaps on disease burden, including in LMICs, need to be addressed to understand the burden and to assess which are the most effective strategies in the future. Understanding these variances will be critical to establishing cost-effective interventions.

There is a need to generate high quality data and ensure dissemination within the scientific community.

There must be engagement with regulators and those responsible for public health system frameworks to build understanding. This will allow evidence-based planning for mAbs introduction in different geographical regions. This introduction will have to pioneer a path into existing organisational or budgetary structures that are designed either for vaccines or therapeutics. Planning for maternal immunisation to protect newborns will be easier within existing systems and may require less lead time but, in countries with poor uptake of existing vaccines, it will be important to have clear paths to overcome obstacles.

Clinicians and researchers will need to find time and capacity to work with regulators, reimbursement authorities, insurers and health system administrators and other health system experts to be sure that appropriate pathways and infrastructures exist for all future technologies.

Given that most hospitalisations due to RSV occur in infants with no prior risk factors, targeting sub-groups may not be the right strategy; a universal campaign may produce better results and require less training and fewer system resources.

Specific strategies are required for the multiple different stakeholders who will be important to assure appropriate use. Particularly critical is that information be made available readily to expectant mothers and their extended families—acceptance among whom will be key to a successful campaign. The list will differ from country to country but most will include obstetricians, gynaecologists and primary care physicians, midwives, advocates for children’s health services and those running groups for people affected by RSV in their families.

As tools are introduced, it will be important that clear frameworks exist for accountability, governance, and responsibility. Acceptance of the recommendations by physicians, nurses and midwives will require programmes of public awareness and education for new parents (including the hygiene measures to prevent RSV), the wider family, and social influencers. This will need to include media and digital channels of communication. Parent support groups, groups run by people affected and health advocacy groups will be indispensable for this effort.

## Conclusion

RSV is an important cause of infant and child disease in developed and developing countries. Novel interventions to prevent RSV in all infants will be available soon. Although new monoclonal antibodies or maternal immunisation may require specific implementation strategies, the overall limited knowledge and awareness of RSV infection is an important hurdle that must be overcome. A multi-stakeholder approach is needed with a central position for parents to ensure readiness in the coming years.

## Data Availability

N/A.

## Abbreviations

ALRI: Acute lower respiratory illness
mAbs: Monoclonal antibodies
MI: Maternal immunization
RSV: Respiratory syncytial virus
RSV-ALRI: RSV-associated acute lower respiratory illness

## References

[1] Shi T, McAllister D, O'Brien K, Simoes E, Madhi S, Gessner B (2017). Global, regional, and national disease burden estimates of acute lower respiratory infections due to respiratory syncytial virus in young children in 2015: a systematic review and modelling study. Lancet.

[2] Griffin MP, Yuan Y, Takas T, Domachowske JB, Madhi SA, Manzoni P (2020). Single-dose nirsevimab for prevention of RSV in preterm infants. N Engl J Med.

[3] Kandeil W, Savic M, Ceregido MA, Guignard A, Kuznetsova A, Mukherjee P (2020). Immune interference (blunting) in the context of maternal immunization with Tdap-containing vaccines: is it a class effect?. Expert Rev Vaccines.

[4] Jarvis JR, Dorey RB, Warricker FDM, Alwan NA, Jones CE (2020). The effectiveness of influenza vaccination in pregnancy in relation to child health outcomes: systematic review and meta-analysis. Vaccine.

[5] Campbell PT, Geard N, Hogan AB (2020). Modelling the household-level impact of a maternal respiratory syncytial virus (RSV) vaccine in a high-income setting. BMC Med.

[6] Lanari M, Prinelli F, Adorni F, Di Santo S, Vandini S, Silvestri M (2015). Risk factors for bronchiolitis hospitalization during the first year of life in a multicenter Italian birth cohort. Ital J Pediatr.

[7] Bozzola E, Ciarlitto C, Guolo S, Brusco C, Cerone G, Antilici L (2020). Respiratory syncytial virus bronchiolitis in infancy: the acute hospitalization cost. Front Pediatr.

[8] Scheltema NM, Gentile A, Lucion F, Nokes DJ, Munywoki PK, Madhi SA (2017). Global respiratory syncytial virus-associated mortality in young children (RSV GOLD): a retrospective case series. Lancet Glob Health.

[9] Pollack P, Groothuis J, Barbarotto G (2002). Development and use of palivizumab (Synagis): a passive immunoprophylactic agent for RSV. J Infect Chemother.

[10] Lozano R, Naghavi M, Foreman K, Lim S, Shibuya K, Aboyans V (2012). Global and regional mortality from 235 causes of death for 20 age groups in 1990 and 2010: a systematic analysis for the Global Burden of Disease Study 2010. Lancet.

[11] Hall C, Weinberg G, Blumkin A, Edwards K, Staat M, Schultz A (2013). Respiratory syncytial virus-associated hospitalizations among children less than 24 months of age. Pediatrics.

[12] Arriola CS, Kim L, Langley G, Anderson EJ, Openo K, Martin AM (2020). Estimated burden of community-onset respiratory syncytial virus-associated hospitalizations among children aged < 2 years in the United States, 2014–15. J Pediatric Infect Dis Soc.

[13] Rha B, Curns A, Lively J, Campbell A, Englund J, Boom J (2020). Respiratory syncytial virus-associated hospitalizations among young children: 2015–2016. Pediatrics.

[14] Pisesky A, Benchimol EI, Wong CA, Hui C, Crowe M, Belair M-A (2016). Incidence of hospitalization for respiratory syncytial virus infection amongst children in Ontario, Canada: a population-based study using validated health administrative data. PLoS ONE.

[15] Descamps A, Launay O, Bonnet C, Blondel B (2019). Seasonal influenza vaccine uptake and vaccine refusal among pregnant women in France: results from a national survey. Hum Vaccin Immunother.

[16] WHO preferred product characteristics for respiratory syncytial virus (RSV) vaccines, initiative for vaccine research (IVR) of the Department of Immunization, Vaccines and Biologicals. 2017. https://apps.who.int/iris/bitstream/handle/10665/258705/WHO-IVB-17.11-eng.pdf. Accessed 8 Oct 2021.

